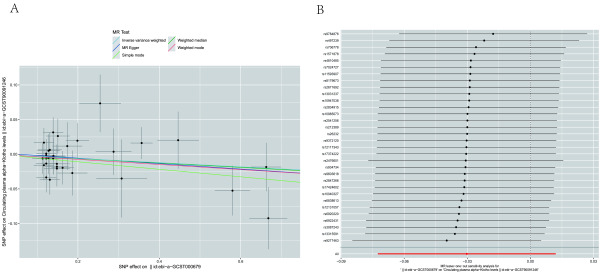# Association between Klotho and autoimmune diseases: A Mendelian randomization study and cross-sectional study

**DOI:** 10.1097/MD.0000000000043788

**Published:** 2025-08-08

**Authors:** Jiayuan Ye, Fengming Wang, Yaojiang Xu, Feihong Xu, Feiqin Shao, Danni Wang

**Affiliations:** a Department of Infectious Diseases, Shangyu People’s Hospital of Shaoxing, Shaoxing University, Shaoxing, Zhejiang Province, China; b Department of Hematopathology, Shangyu People’s Hospital of Shaoxing, Shaoxing University, Shaoxing, Zhejiang, China.

**Keywords:** autoimmune diseases (AIDs), cross-sectional study, Klotho, Mendelian randomization, rheumatoid arthritis (RA)

## Abstract

Klotho is posited to exert a pivotal influence on autoimmune diseases (AIDs). This study endeavors to comprehensively scrutinize the causal relationship between Klotho and 15 AIDs. We employed a two-sample Mendelian randomization (MR) analysis to scrutinize the causal links between Klotho and 15 AIDs. After a rigorous evaluation, potential candidate single-nucleotide polymorphisms (SNPs) for GC and 15 AIDs were extracted from the genome-wide association study dataset. These diseases include rheumatoid arthritis (RA) (2,228,946 SNPs), systemic lupus erythematosus (24,198,877 SNPs), multiple sclerosis (6,304,359 SNPs), Crohn disease (9,457,998 SNPs), celiac disease (518,292 SNPs), ulcerative colitis (24,187,301 SNPs), psoriasis (9,419,702 SNPs), eczema (8,133,670 SNPs), asthma (24,162,338 SNPs), autoimmune hepatitis (24,198,482 SNPs), primary sclerosing cholangitis (7,891,603 SNPs), primary biliary cirrhosis (5,004,018 SNPs), autoimmune thyroid disease (9,419,702 SNPs), type 1 diabetes (59,999,551 SNPs), and pernicious anemia (9419,702 SNPs). Cochran Q value measured heterogeneity, and MR-Egger regression, along with leave-one-out analysis, assessed horizontal pleiotropy. Positive MR outcomes underwent reverse Mendelian analysis and received further validation through cross-sectional studies based on National Health and Nutrition Examination Survey. The MR analysis revealed a significant association: decreased Klotho levels elevate the risk of RA (inverse variance weighting: OR = 0.834, 95% CI = 0.744–0.935, *P* = .0019). However, no such association was found with the risk of the other 14 AIDs. When RA was explored as the exposure, bidirectional associations revealed no genetically predicted links, indicating no causal effects of RA on Klotho (inverse variance weighting: β = −0.030, 95% CI = −0.073 to 0.012, *P* = .161). The cross-sectional findings demonstrated a notable negative correlation between Klotho and RA, aligning with the Mendelian analysis conclusions. Our study revealed a genetically determined association between low Klotho levels and an increased risk of RA, while no causal relationship was observed with the other 14 AIDs. Our cross-sectional study findings aligned with this conclusion. The findings suggest that Klotho levels may serve as a potential predictive indicator for RA, providing a theoretical foundation for utilizing Klotho as a safe and effective means of treating RA.

## 1. Introduction

Autoimmune diseases (AIDs) represent a heterogeneous group of disorders characterized by immune-mediated attacks on the body’s own organs.^[1,2]^ Epidemiological studies have demonstrated the substantial burden imposed by AIDs worldwide. For instance, systemic lupus erythematosus (SLE) posing risks to multiple organs including the kidneys, skin, joints, and nervous system.^[3]^ Rheumatoid arthritis (RA) can lead to cartilage and bone damage, resulting in disability^[4]^ and, in severe cases, serves as a primary contributor to life-altering consequences or mortality.^[5]^ Current treatment approaches for AIDs have limited efficacy and often come with numerous side effects. Therefore, predicting and developing new therapies remains a significant challenge.

Klotho, a novel anti-aging gene encoding a protein with diverse pleiotropic effects, was initially discovered by Kuro-o and colleagues in 1997.^[6–8]^ The Klotho protein exists in 2 forms in the human body: membrane-bound and soluble. Soluble Klotho exerts its biological functions through circulation^[9]^ regulating target tissues by improving oxidative stress, inhibiting inflammation, and modulating cell apoptosis.^[10]^ Previous studies have demonstrated the preventive ability of Klotho against a range of systemic diseases, including chronic kidney disease, interstitial lung disease, and cardiovascular events.^[11–13]^ However, Klotho also exhibits negative regulatory effects, such as cognitive function changes, metabolic disorders, and reduced skeletal muscle mass.^[14–16]^ Immunosenescence is considered a significant risk factor for the development of certain AIDs, associated with functional impairments and decreased immune responsiveness. Klotho plays a crucial role in aging, inflammation, and autoimmunity processes, potentially contributing to the pathogenesis and progression of certain AIDs through multiple mechanisms.^[17]^ Despite epidemiological studies suggesting associations between Klotho and certain AIDs, the causality of these relationships remains to be further explored. Furthermore, these associations derived from traditional observational epidemiological studies are susceptible to confounding factors and reverse causation bias.

Mendelian randomization (MR) is a genetic epidemiological method that utilizes genetic variations as instrumental variables (IVs) to effectively mitigate biases caused by confounding factors and reverse causality. It aims to infer potential causal relationships between exposures and outcomes, and modify the genetic mechanisms of human genetics and complex diseases.^[18,19]^ Additionally, genome-wide association studies (GWAS) have provided robust and reliable IVs for MR research through genetic variation. Allelic variants follow the law of independent assortment, reducing the confounding effects of environmental factors and mimicking the design of randomized controlled trials (RCTs).^[20]^ Recently, causal relationships between various complex diseases, including AIDs, have been widely evaluated using the MR method. Therefore, in our study, we utilized GWAS summary-level data from diverse large-scale cohorts and employed the MR method to determine the causal relationships between Klotho and AIDs (rheumatoid arthritis [RA], systemic lupus erythematosus [SLE], multiple sclerosis [MS], Crohn disease [CD], celiac disease [CeD], ulcerative colitis [UC], psoriasis [PsO], autoimmune hepatitis [AIH], primary sclerosing cholangitis [PSC], primary biliary cirrhosis [PBC], autoimmune thyroid disease [ATD], type 1 diabetes [T1D], pernicious anemia [PA]). Positive results were subsequently subjected to reverse Mendelian analysis and further validated through cross-sectional studies.

## 2. Materials and methods

### 2.1. MR study

#### 2.1.1. Study design

**Figure 1.**
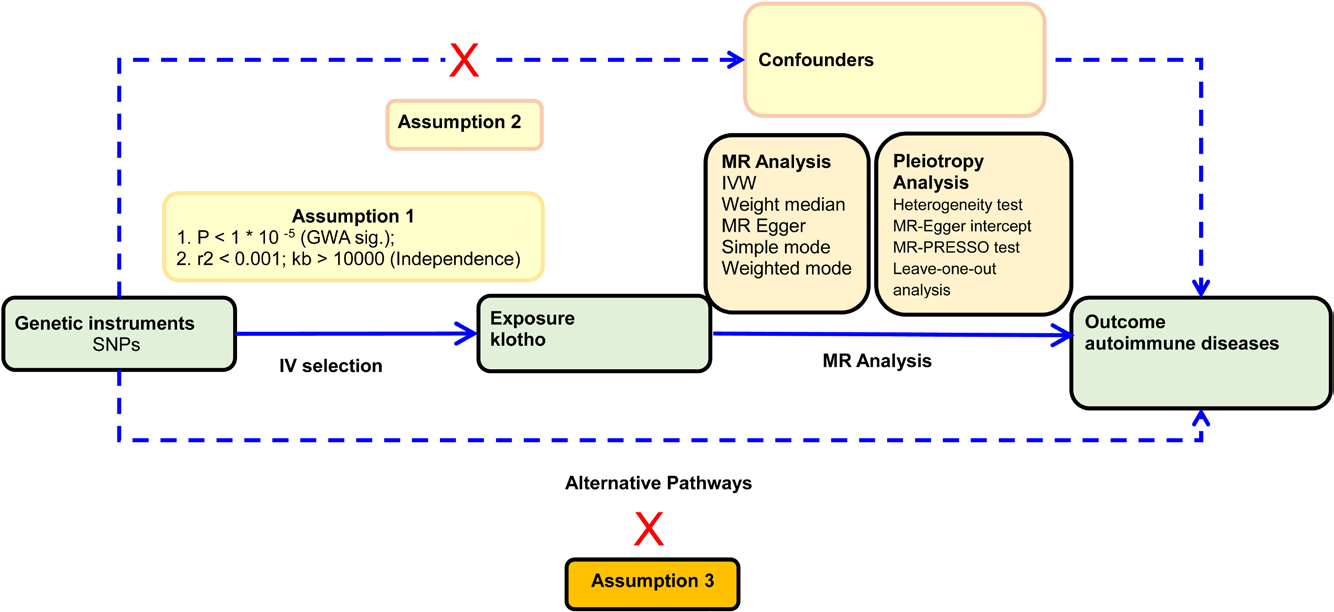
An overview of MR analysis. GWAS = genome-wide association studies, IV = instrumental variable, IVW = inverse variance weighted, MR = Mendelian randomization, MR-PRESSO = MR-Pleiotropy RESidual Sum and Outlier, SNPs = single-nucleotide polymorphisms.

We obtained a dataset of research summary from publicly published studies, all approved by the respective institutions. Since all the data used are from published research and public databases, no additional ethical approval from an institutional review board was required. We employed a two-sample MR analysis to assess the causal relationship between Klotho and 15 AIDs, including RA, SLE, MS, CD, CeD, UC, PsO, eczema, asthma, AIH, PSC, PBC, ATD, T1D, and PA. (Detailed data sources on the exposure can be found in File S1, Supplemental Digital Content, https://links.lww.com/MD/P646.) Klotho was considered as the exposure, while the 15 AIDs served as outcomes. In order to derive reliable causal estimates, the IVs in the MR model must adhere to the 3 assumptions outlined in Figure 1: (1) genetic variants exhibit a significant association with plasma Klotho levels; (2) genetic IVs are not affected by potential confounders; and (3) they exclusively impact the 15 AIDs through plasma Klotho level.

#### 2.1.2. Study cohorts and GWAS

We systematically analyzed GWAS summary-level data from diverse large-scale cohorts to infer the causal relationship between plasma Klotho levels and 15 AIDs. The summary statistics for plasma Klotho levels, involving a sample size of 4376 individuals, can be obtained from the GWAS catalog website (https://gwas.mrcieu.ac.uk/). Additionally, we downloaded the summary statistics for GWASs related to RA, SLE, MS, CD, CeD, UC, eczema, asthma, AIH, PSC, PBC, and T1D from the same GWAS catalog website (https://gwas.mrcieu.ac.uk/) (Table 1). The summary statistics for PsO, ATD, and PA can be obtained from https://www.ebi.ac.uk/gwas/ (Table 1). Notably, all cases and controls in these studies were of European ancestry.

**Table 1:** Characteristics of the klotho and autoimmune disease GWAS cohorts.

Disease	Study	Journal	Cases	Controls	Sample size	Datasets in the GWAS	SNPs	PMID
RA	Stahl EA et al	Nat. Genet.	5539	20,169	25,708	ebi-a-GCST000679	2228946	20453842
SLE	Sakaue S et al	Nat. Genet.	647	482,264	482,911	ebi-a-GCST90018917	24198877	34594039
MS	Patsopoulos NA et al	Science	47,429	68,374	115,803	ieu-b-18	6304359	31604244
CD	de Lange KM	Nat. Genet.	12,194	28,072	40,266	ebi-a-GCST004132	9457998	28067908
CeD	Dubois PC et al	Nat. Genet.	4533	10,750	15,283	ebi-a-GCST000612	518292	20190752
UC	Sakaue S et al	Nat. Genet.	5371	412,561	417,932	ebi-a-GCST90018933	24187301	34594039
PsO	Glanville KP et al	Biol Psychiatry Glob Open Sci.	2759	324,074	326,833	GCST90014457	9419702	34278373
Eczema	Ferreira MA et al	Nat. Genet.	180,129	180,709	360,838	ebi-a-GCST005038	8133670	29083406
Asthma	Sakaue S et al	Nat. Genet.	38,369	411,131	449,500	ebi-a-GCST90018795	24162338	34594039
AIH	Sakaue S et al	Nat. Genet.	821	484,413	485,234	ebi-a-GCST90018785	24198482	34594039
PSC	Ji et al	Nat. Genet.	2871	12,019	14,890	ieu-a-1112	7891603	27992413
PBC	Cordell HJ et al	J Hepatol.	8021	16,489	24,510	ebi-a-GCST90061440	5004018	34033851
ATD	Glanville KP et al	Biol Psychiatry Glob Open Sci.	607	324,074	324,681	GCST90014441	9419702	34278373
T1D	Chiou J et al	Nature	18,942	501,638	520,580	ebi-a-GCST90014023	59999551	34012112
PA	Glanville KP et al	Biol Psychiatry Glob Open Sci.	423	324,074	324,497	GCST90014451	9419702	34278373

AIH = autoimmune hepatitis, ATD = autoimmune thyroid disease, CD = Crohn disease, CeD = celiac disease, GWAS = genome-wide association studies, MS = multiple sclerosis, PA = pernicious anemia, PBC = primary biliary cirrhosis, PSC = primary sclerosing cholangitis, PsO = psoriasis, RA = rheumatoid arthritis, SLE = systemic lupus erythematosus, T1D = type 1 diabetes, UC = ulcerative colitis.

#### 2.1.3. IV selection

We employed the R package TwoSampleMR to meticulously curate genetic instruments from each of the 15 AID GWAS datasets.^[21]^ Our selection comprised independent genetic variants demonstrating significant associations with each exposure (*P* < 1 × 10^−5^) for each instrument. Implementing a clustering procedure with stringent criteria (*R*^2^ < 0.001 and a cluster distance of 10,000 kb), we rigorously excluded any linkage disequilibrium effects.^[22]^ Subsequently, we systematically excluded all ambiguous or palindromic SNPs. In order to discern latent IV bias, we calculated the proportion of variance explained (*R*^2^) and the F-statistic for all SNPs. Instruments surpassing an F-statistic > 10 were deemed suitable for our analyses.^[23,24]^

#### 2.1.4. Statistical analysis

We employed 5 distinct approaches to ensure the credibility of our study results: the inverse variance weighting (IVW) model (if there is heterogeneity, random-effects IVW models are applied; otherwise, the fixed-effect IVW model is applied.), serving as the primary analytical method, along with the MR-Egger, weighted median, weighted mode, and simple mode, all utilized to elucidate potential causal relationships.^[25]^ Subsequently, we quantified heterogeneity using Cochran Q statistic, with a *P*-value < .05 considered indicative of significant heterogeneity.^[26]^ Simultaneously, we employed the intercept term in the MR-Egger regression to assess directional pleiotropy in the causal estimates, confirming significant horizontal pleiotropy when the Egger intercept deviated significantly from 0 and the associated *P*-value was <.05.^[27]^ Furthermore, we conducted MR-Pleiotropy RESidual Sum and Outlier global tests to identify and subsequently exclude any potential outliers introducing horizontal pleiotropy.^[28]^ To bolster the robustness of our findings, we performed sensitivity analyses employing a leave-one-out strategy, iteratively excluding individual SNPs and assessing their impact on the results. All MR analyses were conducted using the “TwoSampleMR” (version 0.5.7, MRC Integrative Epidemiology Unit, Bristol, UK) and “MR-presso” packages in R software version 4.3.0 (Rondo Lab). A *P*-value <.05 was considered indicative of significant statistical differences.

### 2.2. Cross-sectional study

#### 2.2.1. Study design

Based on positive results obtained from MR studies, we conducted a cross-sectional analysis for further validation. The data for the cross-sectional analysis were sourced from the 2007 to 2016 United States National Health and Nutrition Examination Survey (NHANES) database. NHANES is a project aimed at providing objective health data for the U.S. population. All data and methodologies collected by NHANES are accessible for free on the official website (http://www.cdc.gov/nchs/nhanes.htm). The NHANES survey protocol has received approval from the Research Ethics Review Board of the National Center for Health Statistics, and all participants provided informed consent.

**Figure 2.**
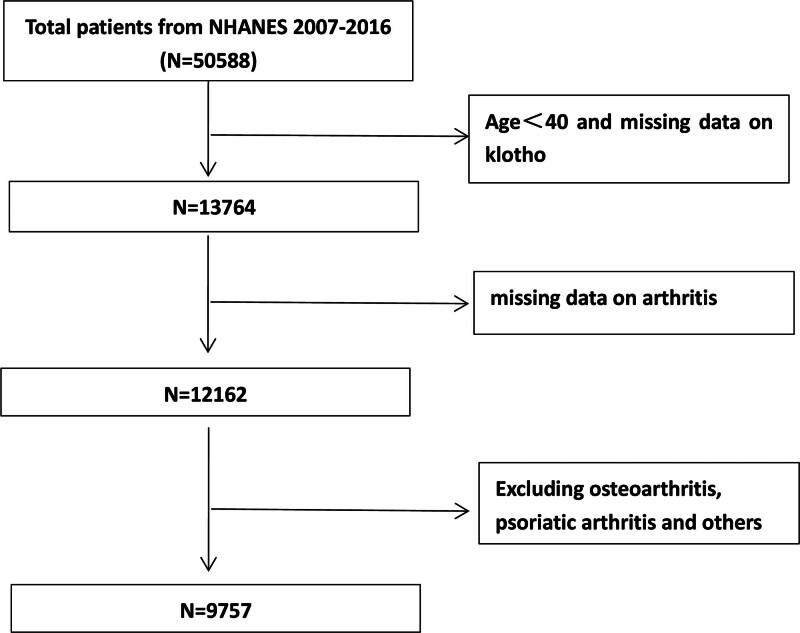
Flow-chart of the study samples.

A total of 50,588 individuals participated in NHANES (2007–2016). We excluded individuals aged < 40 and those lacking klotho level measurements, as well as those who did not complete the arthritis survey. Subsequently, we excluded individuals who responded with “osteoarthritis, psoriatic arthritis, or others” to the question “which type of arthritis was it?” and retained those who answered “rheumatoid arthritis or no” resulting in a final cohort of 9757 individuals (Fig. 2). A prior investigation showcased substantial concordance (85%) between self-reported arthritis and arthritis confirmed through clinical assessment.^[29]^

#### 2.2.2. Measurement of Klotho

Serum samples were stored at –80 °C until analysis using a commercially available Enzyme-Linked Immunosorbent Assay Kit from IBL International, Tokyo, Japan. Duplicate analyses were performed for each sample, and the average of the 2 values was calculated as the final result. Klotho concentrations were also analyzed in duplicate on each Enzyme-Linked Immunosorbent Assay plate, following quality control procedures.

#### 2.2.3. Definition of covariates

Hypertension was defined as meeting any of the following criteria: systolic blood pressure ≥ 140 mm Hg, diastolic blood pressure ≥ 90 mm Hg, self-reported history of hypertension, or current use of antihypertensive medication.^[30]^ Diabetes was diagnosed if any of the following conditions were present: self-reported history of diabetes, current use of antidiabetic medication, glycated hemoglobin level ≥ 6.5%, fasting blood glucose level ≥ 126 mg/dL, or postprandial 2-hour blood glucose level ≥ 200 mg/dL.^[31]^ Physical activity levels were categorized based on metabolic equivalents (MET-min) as < 600, 600 to 1199, or ≥ 1200 MET-min per week. Body mass index (BMI) was calculated as weight divided by the square of height. The estimated glomerular filtration rate (eGFR) was determined using the Chronic Kidney Disease Epidemiology Collaboration equation.^[32]^

#### 2.2.4. Statistical analysis

The analyses were conducted using the R software and EmpowerStats (http://www.empowerstats.com). The Kolmogorov–Smirnov test was applied to assess the normality of the data, which indicated non-normal distributions. For continuous variables with non-normal distributions, the median and interquartile range were reported, and group comparisons were conducted using the Mann–Whitney *U* test. Categorical variables are presented as percentages. We employed multivariable logistic regression analysis to evaluate the relationship between plasma Klotho level and the risk of RA. As a sensitivity analysis, Klotho was categorized into tertiles, and *P*-values for trend tests were calculated. Three models were constructed for this study: Model 1: unadjusted covariates; model 2: adjusted for gender, age, and race; model 3: adjusted for age, gender, race, BMI, eGFR, diabetes status, hypertension status, and physical activity. Ultimately, we utilized a generalized additive model with a spline smoothing function to investigate potential non-linear associations between klotho and the status of RA. A *P*-value < .05 was considered statistically significant.

## 3. Results

### 3.1. MR study

#### 3.1.1. Selection of IVs

After a meticulous screening procedure as previously described, we meticulously chose specific SNPs for our exploration into the causal association between Klotho and 15 AIDs. It was essential to highlight that all these selected SNPs exhibited F-statistics surpassing the threshold of 10, solidifying their role as robust IVs. Comprehensive details regarding these particular SNPs, encompassing their genetic loci and pertinent characteristics, can be located in Table S1, Supplemental Digital Content, https://links.lww.com/MD/P605).

#### 3.1.2. Causal effects of klotho on AIDs

**Figure 3.**
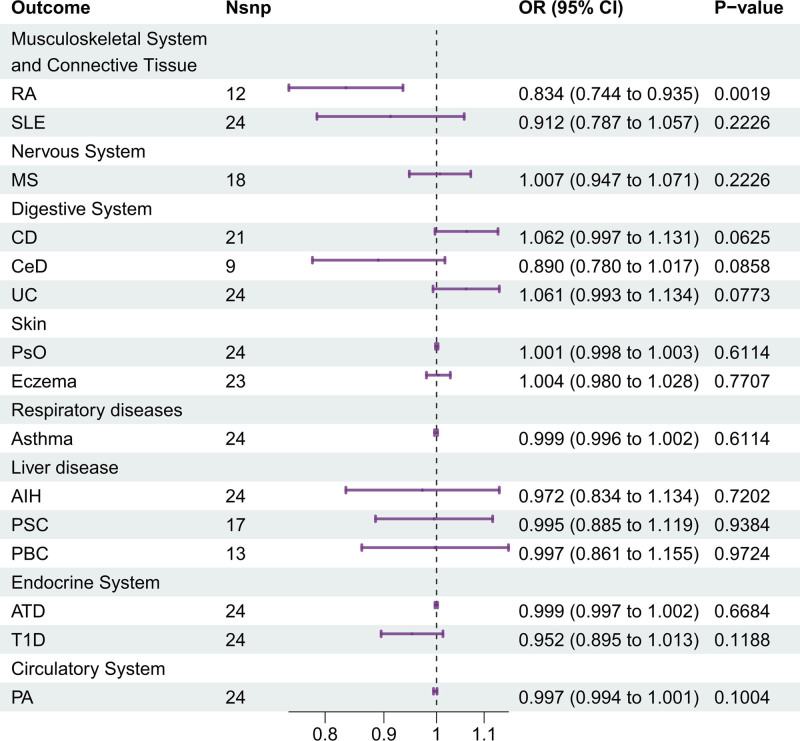
Results of MR analyses between Klotho and autoimmune disease risk. AIH = autoimmune hepatitis, ATD = autoimmune thyroid disease, CD = Crohn disease, CeD = celiac disease, MS = multiple sclerosis, PA = pernicious anemia, PBC = primary biliary cirrhosis, PSC = primary sclerosing cholangitis, PsO = psoriasis, RA = rheumatoid arthritis, SLE = systemic lupus erythematosus, T1D = type 1 diabetes, UC = ulcerative colitis.

The causal relationship between klotho and AIDs from MR analysis was summarized in Figure 3. In the primary IVW MR analysis, higher genetically predicted Klotho was associated with a reduced risk of RA (IVW: OR = 0.834, 95% CI = 0.744, 0.935, *P* = .0019). We also conducted research on other 4 methods, and the findings are as follows: MR-Egger: OR = 0.710, 95% CI = 0.523, 0.963, *P* = .0524; weighted median: OR = 0.786, 95% CI = 0.674, 0.917, *P* = .0022; simple mode: OR = 0.757, 95% CI = 0.587, 0.975, *P* = .0539; weighted mode: OR = 0.768, 95% CI = 0.632, 0.933, *P* = .0220. However, no significant causal association between klotho and the risk of the other 14 AIDs was observed in IVW MR analysis, and this was consistent across the other 4 methods, including MR-Egger, the weighted median, weighted mode and simple mode (all *P* > .05) (Fig. 3 and Table S2, Supplemental Digital Content, https://links.lww.com/MD/P606). Klotho exhibited no significant effect on SLE (IVW: OR = 0.912, 95% CI = 0.787–1.057, *P* = .2226), MS (IVW: OR = 1.007, 95% CI = 0.947–1.071, *P* = .2226), CD (IVW: OR = 1.062, 95% CI = 0.997–1.131, *P* = .0625), CeD (IVW: OR = 0.890, 95% CI = 0.780–1.017, *P* = .0858), UC (IVW: OR = 1.061, 95% CI = 0.993–1.134, *P* = .0773), PsO (IVW: OR = 1.001, 95% CI = 0.998–1.003, *P* = .6114), eczema (IVW: OR = 1.004, 95% CI = 0.980–1.028, *P* = .7707), asthma (IVW: OR = 0.999, 95% CI = 0.996–1.002, *P* = .6114), AIH (IVW: OR = 0.972, 95% CI = 0.834–1.134, *P* = .7202), PSC (IVW: OR = 0.995, 95% CI = 0.885–1.119, *P* = .9384), PBC (IVW: OR = 0.997, 95% CI = 0.861–1.155, *P* = .9724), ATD (IVW: OR = 0.999, 95% CI = 0.997–1.002, *P* = .6684), T1D (IVW: OR = 0.952, 95% CI = 0.895–1.013, *P* = .1188), or PA (IVW: OR = 0.997, 95% CI = 0.994–1.001, *P* = .1004) (Fig. 3).

#### 3.1.3. Sensitivity analysis

**Figure 4.**
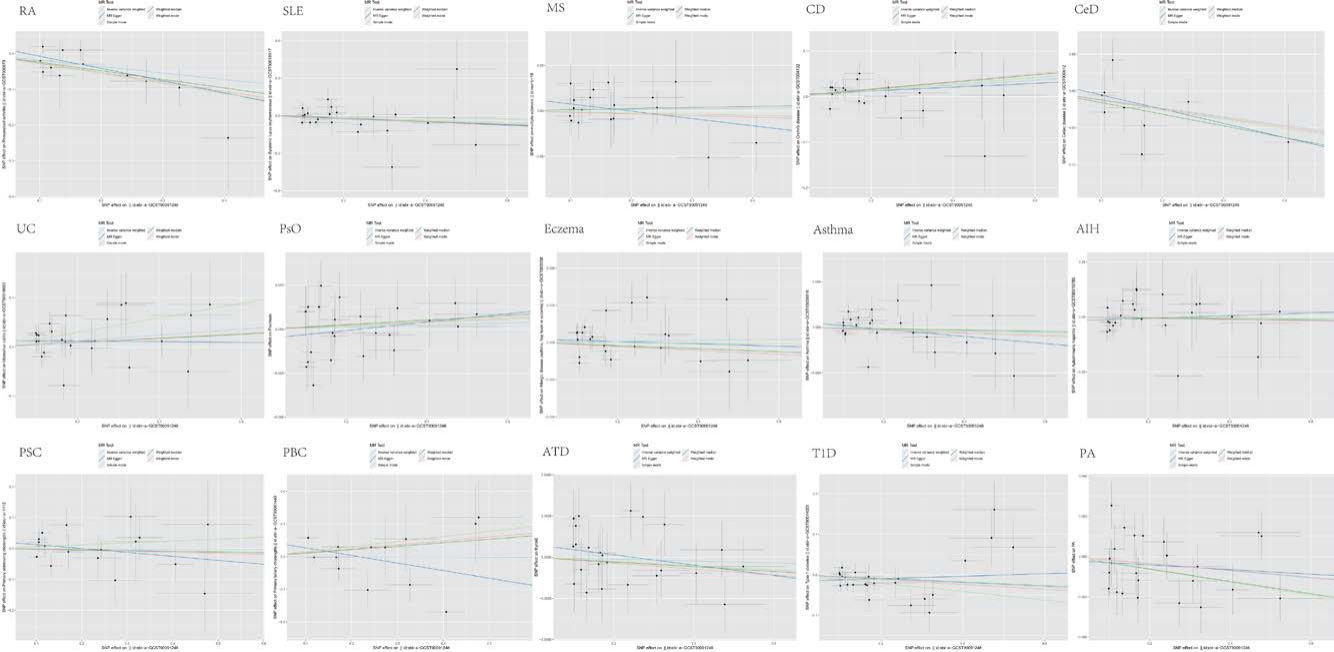
Scatter plots show the MR effect of Klotho on each autoimmune diseases in different MR methods. MR = Mendelian randomization.

**Figure 5.**
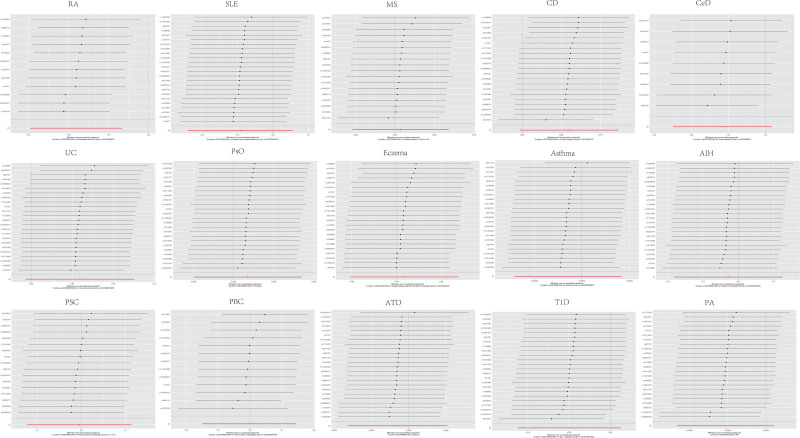
Leave-one-out plots for the causal relationship between Klotho and autoimmune diseases.

Heterogeneity was not detected in most AID types based on Cochran Q test for Klotho IVs; however, substantial heterogeneity was observed for asthma (IVW: Q = 40.312; *P* = .014) and PBC (Q = 25.458; *P* = .013) (Table 2). In the MR analysis of Klotho on AIDs, MR-Egger regression intercepts for all AIDs did not deviate from the null value, suggesting the absence of horizontal pleiotropy (all intercept *P*-values > .05) (Table 2). The MR-Pleiotropy RESidual Sum and Outlier test identified 1 outlier (rs9411378) for asthma but none for PBC. Upon outlier removal, heterogeneity disappeared for asthma (IVW: Q = 22.458; *P* = .433), but no significant effect on klotho for asthma was observed (outlier-corrected IVW: OR = 1.000; 95% CI = 0.998–1.002; *P* = .7988). Scatter plots illustrating the causal associations from MR analysis are depicted in Figure 4. Leave-one-out analysis confirmed that no single SNP was driving the observed causal effects (Fig. 5).

**Table 2:** Sensitivity analysis between Klotho and autoimmune disease risk.

Outcome	Cochran Q		MR-Egger		MR-PRESSO global outlier test
	Q value	Q_pval	Intercept	P	Outlier
RA	11.953	0.367	0.027	0.291	None
SLE	19.805	0.654	0.010	0.725	None
MS	12.070	0.796	0.016	0.219	None
CD	25.386	0.187	0.003	0.796	None
CeD	6.972	0.540	0.014	0.599	None
UC	25.607	0.320	0.013	0.311	None
PsO	21.805	0.532	-0.001	0.376	None
eczema	33.708	0.053	0.002	0.656	None
Asthma	40.312	0.014	0.000	0.425	rs9411378
AIH	15.941	0.858	-0.018	0.545	None
PSC	14.412	0.568	0.025	0.292	None
PBC	25.458	0.013	0.050	0.172	None
ATD	19.204	0.689	0.001	0.297	None
T1D	34.531	0.058	-0.015	0.205	None
PA	31.772	0.105	0.000	0.753	None

AIH = autoimmune hepatitis, ATD = autoimmune thyroid disease, CD = Crohn disease, CeD = celiac disease, MS = multiple sclerosis, PA = pernicious anemia, PBC = primary biliary cirrhosis, PSC = primary sclerosing cholangitis, PsO = psoriasis, RA = rheumatoid arthritis, SLE = systemic lupus erythematosus, T1D = type 1 diabetes, UC = ulcerative colitis.

#### 3.1.4. Causal effects of RA on Klotho

When RA was considered as the exposure to explore bidirectional associations, no significant genetically predicted associations were observed (Tables S3 and S4, Supplemental Digital Content, https://links.lww.com/MD/P606 and Figure S1, Supplemental Digital Content, https://links.lww.com/MD/P604, indicating the absence of causal effects of RA on Klotho (IVW: β = −0.030, 95% CI = −0.073–0.012, *P* = .161).

### 3.2. Cross-sectional study for Klotho and RA

Based on the MR analysis results mentioned above, Klotho was only causally associated with a higher risk of RA. Therefore, we conducted a cross-sectional analysis based on the NHANES database as another sensitivity analysis to further validate the relationship between Klotho and RA.

**Figure 6.**
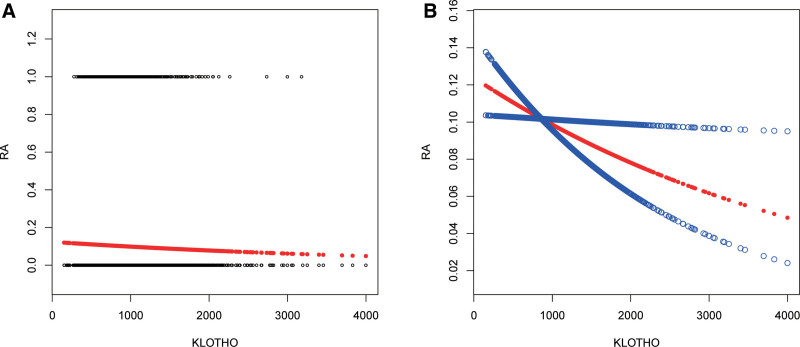
Non-linear association between the klotho and RA in adults. (A) Each black point represents a sample. (B) Solid redline represents the smooth curve fit between variables. The dashed lines are 95% confidence intervals. gender, age, and race were adjusted. Model 3: gender, age, race, BMI, hypertension, diabetes, physical activity, cotinine and eGFR were adjusted. BMI = body mass index, eGFR = estimated glomerular filtration rate, RA = rheumatoid arthritis.

Baseline characteristics of 9757 adult participants were detailed in Table 3 based on RA status. Participants with RA exhibited lower Klotho levels compared to non-RA counterparts (*P* = .026). Additionally, those with RA were older, more frequently women, predominantly non-Hispanic Black, had higher BMI, and lower eGFR. They displayed a higher prevalence of hypertension and DM. Subsequently, multivariate regression analysis was conducted to assess the association between klotho and RA prevalence (Table 4). Klotho demonstrated a negative correlation with RA status across all 3 models (model 1: OR = 0.924, 95% CI: 0.862–0.991; model 2: OR = 0.923, 95% CI: 0.862–0.990; model 3: OR = 0.923, 95% CI: 0.861–0.991). When Klotho was transformed from a continuous variable to a categorical variable (tertiles), participants in tertiles 2 and tertiles 3 exhibited a 11.7% and 15.5% reduced risk of RA, respectively, compared to tertiles 1 in model 3. A significant linear trend was evident for the correlation between Klotho tertiles and RA status (model 1: *P* = .012; model 2: *P* = .043; model 3: *P* = .047). These findings indicated that individuals with elevated klotho levels were less prone to developing RA. Furthermore, this negative correlation persisted in smooth curve fittings and generalized additive models (Fig. 6).

**Table 3:** Weighted characteristics of the RA and non-RA groups.

	Total (n = 9757)	Non-RA (n = 8764)	RA (n = 993)	*P*-value
Klotho (pg/mL), mean (Q₁, Q₃)	806.600 (661.600–1002.100)	810.250 (664.075–1003.825)	782.800 (642.900–985.500)	.026
Age (years), mean (Q₁, Q₃)	55.000 (47.000–64.000)	54.000 (46.000–63.000)	61.000 (52.000–68.000)	<.001
Gender (%)				<.001
Male	5053 (51.788%)	4651 (53.069%)	402 (40.483%)	
Female	4704 (48.212%)	4113 (46.931%)	591 (59.517%)	
Race (%)				<.001
Non-Hispanic White	3819 (39.141%)	3449 (39.354%)	370 (37.261%)	
Non-Hispanic Black	1947 (19.955%)	1648 (18.804%)	299 (30.111%)	
Hispanic	1700 (17.423%)	1538 (17.549%)	162 (16.314%)	
Other race	2291 (23.481%)	2129 (24.293%)	162 (16.314%)	
BMI (kg/m^2^), mean (Q₁, Q₃)	28.220 (24.800–32.160)	28.000 (24.700–31.800)	30.500 (26.500–35.210)	<.001
eGFR, mL/(min × 1.73 m^2^) (%), mean (Q₁, Q₃)	91.418 (76.891–103.276)	91.869 (77.723–103.586)	87.302 (69.714–99.941)	<.001
Hypertension (%)				<.001
No	4994 (51.184%)	4689 (53.503%)	305 (30.715%)	
Yes	4763 (48.816%)	4075 (46.497%)	688 (69.285%)	
Diabetes (%)				<.001
No	7446 (76.314%)	6810 (77.704%)	636 (64.048%)	
Yes	2303 (23.604%)	1946 (22.204%)	357 (35.952%)	
Not recorded	8 (0.082%)	8 (0.091%)	0 (0.000%)	
Physical activity, MET time/week (%)				<.001
<600	1416 (14.513%)	1273 (14.525%)	143 (14.401%)	
600–1199	1100 (11.274%)	1004 (11.456%)	96 (9.668%)	
≥1200	4518 (46.305%)	4150 (47.353%)	368 (37.059%)	
Not recorded	2723 (27.908%)	2337 (26.666%)	386 (38.872%)	

Non-normally distributed continuous variables: median (interquartile range); categorical variables: percentages.

BMI = body mass index, eGFR = estimated glomerular filtration rate, RA = rheumatoid arthritis.

**Table 4:** Odds ratio (OR) for RA status based on Klotho.

	Model 1 OR (95% CI)	*P*	Model 2 OR (95% CI)	*P*	Model 3 OR (95% CI)	*P*
Klotho (per SD increase)	0.924 (0.862, 0.991)	.026	0.923 (0.862, 0.990)	.024	0.923 (0.861, 0.991)	.027
Q1 (151.30–709.20)	Reference		Reference		Reference	
Q2 (709.30–926.90)	0.807 (0.688, 0.946)	.008	0.869 (0.738, 1.023)	.091	0.883 (0.748, 1.042)	.141
Q3 (927.00–3998.50)	0.818 (0.698, 0.959)	.013	0.846 (0.718, 0.997)	.045	0.845 (0.715, 0.999)	.049
*P* for trend	.012		.043		.047	

Model 1: unadjusted covariates; model 2: adjusted for gender, age, and race; model 3: adjusted for age, gender, race, BMI, eGFR, diabetes status, hypertension status, and physical activity.

BMI = body mass index, eGFR = estimated glomerular filtration rate, RA = rheumatoid arthritis.

## 4. Discussion

In this investigation, we employed a two-sample MR approach to explore the causal association between Klotho and 15 AIDs. Our findings revealed a causal link between low Klotho levels and an elevated risk of RA, while no significant causal relationship was identified with the other 14 AIDs. Expanding on these favorable results, we conducted a reverse Mendelian analysis on the RA–Klotho association, indicating no reverse causal link between RA and Klotho. Subsequently, we conducted an additional sensitivity analysis through a cross-sectional study using NHANES database (2017–2016) to further investigate the relationship between Klotho and RA. The results indicated a significant negative correlation between Klotho and RA, consistent with the conclusions drawn from the Mendelian analysis. To our knowledge, this is the first research to systematically investigate the causal relationship between Klotho and 15 different AIDs.

AIDs are characterized by pathological states in which abnormal immune responses targeting normal body components lead to inflammation, cellular damage or dysfunction, and clinical manifestations.^[33,34]^ Their development depends on the imbalance between pathogenic factors produced by self-reactive T cells and B cells and the regulatory factors that normally control immune responses. Despite varying etiologies, AIDs exhibit certain common mechanisms of pathogenesis.^[35]^ Immunological plasticity is crucial for maintaining immune homeostasis, and the aging of the immune system is associated with the inability of the body to recognize self-antigens, often resulting in autoimmune reactions.^[17,36]^ The hallmark of immunosenescence is the decline of the lymphocyte pool, including B and T cells, and subsequent immune dysregulation.^[37,38]^

Klotho is a beta-glucuronidase associated with the mechanisms of aging.^[39]^ The full-length membrane form of the protein consists of a large extracellular domain, a transmembrane segment, and a short intracellular domain.^[40]^ Studies have shown that high expression of the Klotho gene can extend the lifespan of mice and enhance their antioxidant stress capacity, indicating that Klotho is a gene with anti-apoptotic and anti-aging effects.^[8,41]^ As an anti-aging gene, Klotho is believed to be associated with AIDs. Some studies have investigated the relationship between Klotho and certain AIDs, finding no association between Klotho and MS,^[42,43]^ as well as no association between Klotho and SLE.^[44]^ However, it has been found that Klotho is significantly decreased in T1D patients and may be related to other factors such as the kidneys.^[45]^ The relationship between Klotho and other AIDs remains unclear. Therefore, we analyzed the relationship between Klotho and 15 AIDs to explore their associations. Our study revealed that Klotho exhibits a negative causal relationship only with RA, while no causal relationship was observed with the other 14 AIDs.

RA is a common systemic AID characterized by chronic systemic inflammation and symmetrical inflammatory polyarthritis of unknown etiology, with an incidence of approximately 1% in the general population.^[46]^ The pathogenesis of RA is complex and influenced by multiple genetic, environmental, immune, and other factors that affect disease occurrence, development, and presentation.^[47,48]^ RA is caused by the formation of antibodies stimulated by numerous joint-specific native or modified antigens, whose peptides are recognized by T cells involved in synovial inflammation, resulting in subsequent activation of self-antibody-producing B cells or Th17 and other Th cell subsets.^[49]^ During the process of T-cell activation following antigen stimulation, co-stimulation is an important step, which is likely to play a critical role in the pathogenesis of RA synovitis.^[50,51]^ Other cytokines such as TNF,^[52,53]^ the IL-1 family members (IL-1, IL-18, and IL-33),^[54–56]^ the typical Th17 cytokine IL-17,^[57,58]^ and the IL-6 family, including IL-6 and leukemia inhibitory factor,^[59,60]^ all play important roles in causing inflammation, joint destruction, and RA-related complications. In addition, significant research has shown that peripheral blood CD4 + lymphocytes in RA patients are similar to lymphocytes undergoing physiological aging, which may indicate that RA leads to accelerated aging of T-cell populations.^[61,62]^ Studies have found that Klotho mRNA expression levels in human CD4 + lymphocytes decrease significantly with age, and in CD4 + cells of RA patients, Klotho expression is severely suppressed regardless of age.^[63]^ Furthermore, Klotho reversely regulates NFκ-B and reduces pro-inflammatory gene transduction, which may be related to the pathogenesis of RA.^[64]^

Currently, there are limited studies examining the relationship between Klotho and RA. In 2 retrospective studies with small sample sizes (128 patients and 80 patients, respectively), Klotho levels were found to be higher in RA patients compared to healthy individuals, particularly among those undergoing treatment.^[65,66]^ These studies suggested a compensatory increase in Klotho protein levels in RA patients. However, our reverse Mendelian analysis of RA and Klotho did not reveal a genetically causal relationship between RA and Klotho. In this study, we expanded our participant pool to a larger-sample size and conducted a more comprehensive assessment by combining MR analysis with cross-sectional research. Our Mendelian analysis revealed a genetic-level association, indicating that individuals with elevated Klotho levels have a reduced risk of RA. Furthermore, we validated this observation through an extensive cross-sectional analysis, employing multivariable logistic regression, smooth curve fittings, and generalized additive models. Our findings align with the conclusions drawn from MR analysis. Thus, this discovery holds significant implications for public health, suggesting that Klotho not only has potential as a predictive marker for RA risk but also as a promising avenue for the development of a safe and effective therapeutic approach.

The primary strength of this study lies in the integration of a two-sample MR analysis and a large-scale cross-sectional survey based on NHANES. Cross-sectional studies can facilitate epidemiological explorations, while MR analysis can alleviate the impact of inherent residual confounding, reverse causation, and measurement errors in traditional epidemiological investigations. However, the study also has several limitations. Firstly, the cross-sectional and genetic data were sourced from different racial populations, with the MR study sample comprising individuals of European ancestry, while the cross-sectional study predominantly included Americans. To mitigate potential confounding related to race, future research should prioritize studying homogeneous racial groups. Secondly, despite adjusting for crucial covariates in the cross-sectional study, the presence of other factors, such as drug use, may have introduced bias. Additionally, the diagnosis of RA relied on self-reporting, and self-reported confounders might be susceptible to bias. Finally, despite efforts to exclude outlier variants, the influence of pleiotropy on the results cannot be entirely dismissed. Therefore, larger-sample MR studies or RCTs are essential for obtaining more precise and reliable results.

In summary, our study reveals a genetic link between low Klotho levels and heightened RA risk via MR analysis, distinct from 14 other AIDs. Supported by our cross-sectional study, Klotho emerges as a promising predictive biomarker for RA and a potential therapeutic target. Further exploration is warranted to elucidate Klotho impact on RA onset and progression, requiring validation through large-scale RCTs and rigorous animal studies.

## Acknowledgments

The authors thank the NHANES and the authors and participants of the involved GWAS for providing summary statistics data.

## Author contributions

**Conceptualization:** Jiayuan Ye.

**Data curation:** Jiayuan Ye, Fengming Wang, Yaojiang Xu, Feihong Xu, Feiqin Shao, Danni Wang.

**Formal analysis:** Jiayuan Ye.

**Writing – original draft:** Jiayuan Ye, Yaojiang Xu.

**Writing – review & editing:** Jiayuan Ye.

## Supplementary Material



**Figure:**