# Supplementary material for: Association between Klotho and autoimmune diseases: A Mendelian randomization study and cross-sectional study
Source: Medicine (Baltimore). 2025 Aug 8;104(32):e43788. doi: 10.1097/MD.0000000000043788 (PMC12338200; doi:10.1097/MD.0000000000043788)
Supplement: Supplementary file 1 [file medi-104-e43788-s001.docx]

1. RA：The exposure source comes from a genome-wide association study meta-analysis conducted on 5,539 rheumatoid arthritis individuals with autoantibodies (cases) and 20,169 controls of European ancestry.
2. MS：The exposure source comes from 1,864 UK cases, 1,400 US cases, 794 AUS cases, 2,246 CE cases, 950 MEDI cases, 583 FINLAND cases, and 1,976 NORDIC cases of MS patients.
3. CD：The exposure source comes from our GWAS conducted on 12,160 IBD cases and 13,145 controls of European ancestry.
4. CeD： The exposure source comes from five European celiac disease case and control sample collections, including 4,533 celiac disease cases and 10,750 individuals of European ancestry.
5. PsO、ATD、PA：The exposure source comes from the UKB, a prospective health study involving 500,000 individuals in the United Kingdom.
6. Eczema： The exposure source comes from 180,129 individuals of European ancestry who reported having asthma and/or hay fever and/or eczema.
7. PSC：The exposure source comes from 731 PSC cases from the Scandinavian Peninsula and Germany, 1,227 UK PSC cases, and a group of 904 US PSC patients.
8. PBC：The exposure source comes from 10,516 individuals with PBC and 20,772 healthy individuals recruited in Canada, China, Italy, Japan, the UK, or the USA.
9. T1D：The exposure source comes from a GWAS conducted on 18,942 T1D cases and 501,638 controls of European ancestry from 9 cohorts.
10. SLE、UC、Asthma、AIH：The exposure source comes from 220 deep-phenotype genome-wide association studies conducted in BioBank Japan (n = 179,000), incorporating past medical history and text-mining of electronic medical records.
